# Diazoxide-unresponsive congenital hyperinsulinism associated with ABCC8 nonsense mutation

**DOI:** 10.1186/1687-9856-2015-S1-P86

**Published:** 2015-04-28

**Authors:** Suhaimi Hussain, Sian Ellard, Sarah Flanagan

**Affiliations:** 1 Paediatric Department School of Medical Sciences Hospital University Science Malaysia, Kota Bharu, Kelantan, Malaysia; 2 Molecular Genetics Laboratory, Royal Devon and Exeter NHS Healthcare, Exeter, UK

## Background

Congenital hyperinsulinism is a dysregulated insulin secretion that results in persistent hypoglycemia. It is a heterogenous condition but common to all is the inappropriately high insulin during hypoglycemia with absence ketone bodies and reduced free fatty acids.

## Case presentation

A baby boy with birth weight of 2.4kg at 35 weeks was born via Caesarian section. The boy was allowed feeding on demands, however he had the first onset of hypoglycemia at 2 hours of life. His blood sugar ranged from low reading to 2.5 mmol/L. The patient was treated with boluses of intravenous dextrose D10% followed by maintenance dextrose with its increasing strength in order to treat the refractory hypoglycemia. In addition to that intravenous hydrocortisone and glucagon infusion were started. The patient’s blood sugar could only be maintained > 3.0mmol/L after a glucose load of 30mg/kg/min and a glucagon infusion of 50mcg/kg/hour. During hypoglycemia, the insulin level was 19.6 pmol/L (17.8-173.0) and blood ketone was negative. Oral diazoxide was started at 5.0mg/kg/day in divided doses combined with chlorothiazide 7.0 mg/kg/day. Diazoxide was titrated up to 20.0mg/kg/day as he had a poor response even after 1 week of the treatment. Apart from that oral nifidipine 2.5 mg/kg/day in divided doses was also started after a few days with the combination therapy. Only after starting octreotide infusion a good rise of blood sugar was seen within 1 hour and the glucose load could be brought down and the other drugs were off. The boy was discharged with subcutaneous octreotide infusion at 2mcg/hour via portable insulin pump.

## Results

The boy is heterozygous for an ABCC8 nonsense mutation, p.R934*. A second ABCC8 mutation has not been found and sequencing of the KCNJ11 gene failed to detect a change from the normal sequence. He has inherited the mutation from his father; a focal lesion is therefore possible. 18F-DOPA PET-CT scanning is recommended and if a focal lesion is identified and surgically resected, microsatellite analysis of the DNA can be undertaken to confirm loss of heterozygosity.

## Conclusion

KATP mutation is the commonest cause for diazoxide resistant congenital hyperinsulinism.

Continuous subcutaneous octreotide infusion is a feasible alternative to pancreatic surgery.

Written informed consent was obtained from the patient's parent or guardian for publication of this abstract and any accompanying images. A copy of the written consent is available for review by the Editor of this journal.

